# Successful management of critical iliac artery aneurysm which is unexpectedly accompanied by acute aortic dissection type B: A case report

**DOI:** 10.1002/ccr3.1807

**Published:** 2018-09-12

**Authors:** Toktam Alirezaei, Mohamad Mozafar

**Affiliations:** ^1^ Cardiology Department of Shohaday‐e‐Tajrish Hospital SBMU Tehran Iran; ^2^ Vascular Surgery Department of Shohaday‐e‐Tajrish Hospital SBMU Tehran Iran

**Keywords:** aorta, aortic aneurysm, large common iliac aneurysm, type B aortic dissection

## Abstract

Acute aortic dissection with concurrent large aortic aneurysm is a catastrophic condition. Clinicians may be faced with the dilemma of how to manage these patients. This case reports a successful management crisis in a patient with a type B aortic dissection and a large left common iliac artery aneurysm.

## 1 INTRODUCTION

Aortic dissection is the main and common catastrophe of the aorta which requires immediate treatment. The reported mortality rate of acute aortic dissection increases at the rate of 1%‐2% per hour when left untreated.^1^ Without any treatment, about 33% of patients die inside the first 24 hours, and 50% die just under 48 hours.^2^, ^3^

An abdominal aortic aneurysm is a considerable burden on health care system and its occurrence has shown an increase during the past two decades. The risk of rupture in large aortic aneurysms is very high and the first goal should be to prevent this type of rupture in the patient health supervision.^4^, ^5^

While, management of the aortic dissection or a large aortic aneurysm, each one, is difficult and despite advances in diagnostic and therapeutic modalities it is associated with a high mortality rate, it is common for clinicians to be faced with the question, how to manage patients with acute aortic dissection with simultaneous critical aortic aneurysm in life‐threatening setting, which may be catastrophic in the event of mismanagement or treatment delay.

## 2 CASE REPORT

A 51‐year‐ old male waspresented to an outside hospital with the complaint of bilateral flank pain radiating to the abdomen that started the day before. The pain was generalized, persistent, and not relevance to the position while the patient denied having nausea, vomiting, or diarrhea.

The patient had a past medical history of hypertension started 5 years ago and his medication included losartan 50 mg daily but he has not being taking it regularly. This patient had a 35 pack‐year smoking history.

On initial evaluation, his vital signs were a blood pressure of 230/120 mm Hg without significant difference between two arms, a heart rate of 95 beats per minute, a respiratory rate of 20 per minute with an oxygen saturation of 94% breathing air. A cardiac exam showed no murmur. Peripheral pulses were intact and symmetrical.

Pulmonary findings consisted of decreased breath sound at the base of the left lung. Patience abdomen was soft, without pulsatile mass, and no tenderness was present.

**Figure 1:**
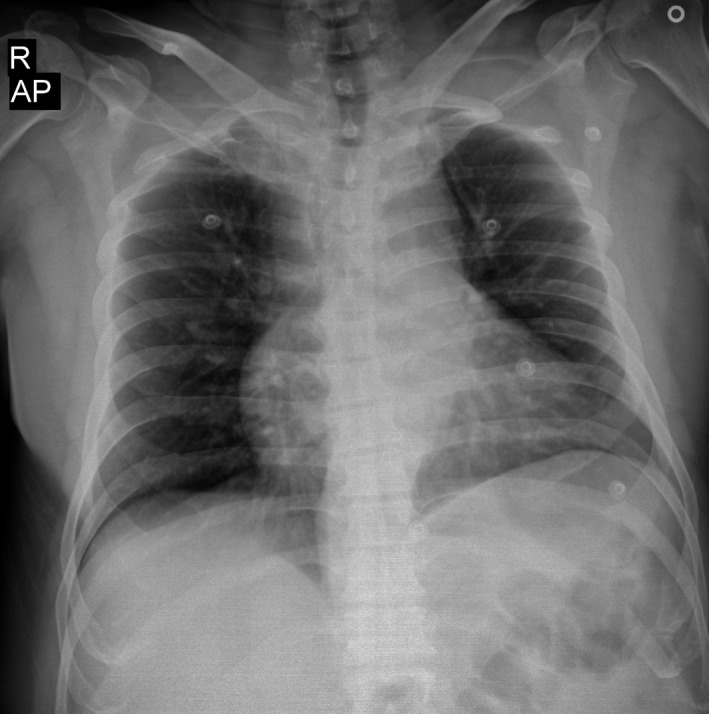
Chest radiograph demonstrating a widened mediastinum

The chest radiograph showed mild mediastinal widening with no active lung lesions (Figure 1).

Electrocardiogram (EKG) was taken and showed normal sinus rhythm with nonspecific ST deviation.

Due to complain of flank pain, sonographic evaluation was performed and despite limited value within the pelvis caused by gas and obesity, suggested the presence of an iliac artery aneurysm measuring 6 cm with no evidence of rupture.

Decision was made to transfer this patient to our emergency department for further evaluation. Upon arrival to our institution, the patient's blood pressure showed 210/120 mm Hg in both arms and the heart rate was 90 beats per minute. The peripheral pulses were equal, bilateral, and full. Sensory and motor testing were unremarkable.

The laboratory findings, including cardiac enzyme levels, glucose, hemoglobin, were all within normal limits and nothing specific was found on the laboratory tests except a creatinine: 1.6 mg/dL.

**Figure 2:**
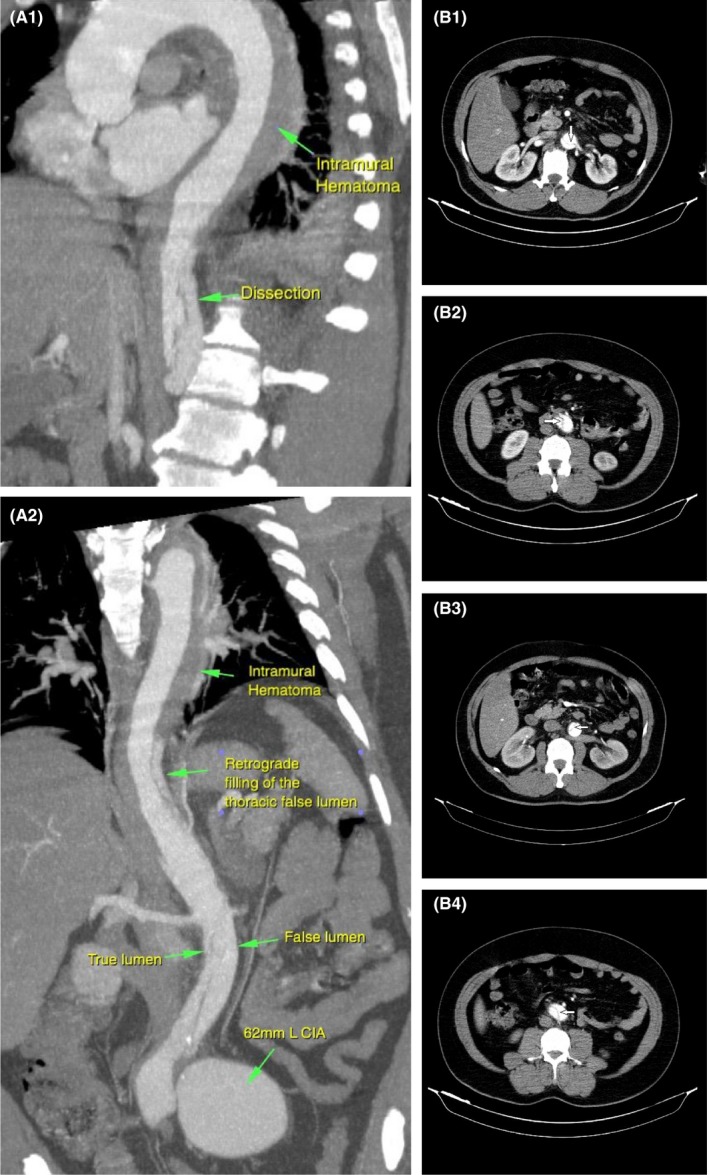
A, Aortic CT Angiography showing Type B aortic intramural hematoma & classic aortic dissection and left common iliac artery aneurysm. B, Aortic CT Angiography demonstrating Type B acute aortic dissection (arrows)

In our institution, aortic CTA was performed to determine which treatment should be used. The result of the CTA of aorta demonstrated a large left common iliac artery aneurysm (62 mm in diameter), unpredictably, other finding in CT was classic aortic dissection type B starting from the distal of thoracic aorta right above the diaphragm with its distal extent to the iliac artery that appeared to be complicated with intramural hematoma type B starting from the distal of aortic arch to the site of dissection (Figures 2A,B).

Initial therapy prescription was to include two large‐bore intravenous lines (IVs), oxygen, respiratory monitoring, and monitoring of cardiac rhythm, blood pressure, and urine output, stabilizing the patient and controlling pain. Clinically, the patient was assessed frequently for hemodynamic compromise, mental status changes, neurologic, or peripheral vascular changes.

Following multidisciplinary assessments, consensus was achieved. In event of encountering simultaneous acute aortic dissection and large common iliac artery aneurysm, the very starting act is to pay serious attention to medical treatment of type B aortic dissection with careful monitoring for the development of complications and close monitoring of visceral and extremity perfusion and renal function. Nevertheless, if there is an extant or impending complication of aortic dissection or aortic aneurysm, think through an immediate surgical or endovascular intervention, otherwise, the procedure for aortic aneurysm is delayed.

The patient was monitored in the cardiac intensive care unit and medical treatment was started aggressively through management of heart rate and blood pressure with the goal of reaching heart rate 60 bpm and systolic blood pressure 100‐120 mm Hg. He received an intravenous labetalol bolus as first‐line agents followed by continuous infusions of intravenous nitroglycerin (because nitroprusside was not available). Due to reno vascular hypertension that is caused by extension of the dissection flap to the renal arteries, lowering blood pressure was very difficult and despite the infusions of labetalol and esmolol and nitroglycerin titrated to max dose, blood pressure reached 170/110.

Finally, after administration of several doses of intravenous propranolol, heart rate was controlled between 60‐70 beats per minute and systolic blood pressure reached 110/80 mm Hg.

After the control of heart rate and blood pressure, the patient was stable and relieved of pain. After multidisciplinary consultation, the decision was made to do an operation on the eighth day of admission, and after proximal and distal control of aneurysm.

The operation was done with a midline incision and after control of the proximal and distal segments of the aneurysm, endoaneurysmorraphy was done with a tubular graft of Dacron 10f. Postoperative period, the patient continued to show clinical improvement. He got transitioned to oral medications to control the heart rate and blood pressure (Metoprolol, diltiazem, valsartan, amlodipine, hydrochlorothiazide).

The remainder of the hospital course was not complicated and the patient was discharged on hospital day 18. During the 18 month's follow‐up and review, the patient stopped cigarette smoking and was compliant with a stable regimen of valsartan, metoprolol, hydrochlorothiazide, and amlodipine, which lead to maintain normal blood pressure.

## 3 DISCUSSION

This study case reports a 51‐year‐old man displaying bilateral flank pain that was thought to be from a large aortic aneurysm, although later diagnosed as a concurrent acute aortic dissection type B. According to Hagan et al^6^, up to 30% of patients who have later diagnosed with aortic dissection during the initial evaluation when suspected of having other situations, such as aneurysms, ACSs, aortic stenosis and pericarditis.

Aortic dissection is a catastrophic life‐threatening condition, caused by a separation of the layers within the aortic wall.^7^ Tears in the intimal layer result in the propagation of dissection (proximally or distally) secondary to blood entering the intima‐media space.^8^

The Stanford classification of aortic dissection divides them into two types, type A and type B. Type A involves the ascending aorta and Type B does not involve the ascending aorta.^9^ This system helps to delineate treatment. Usually, type A dissections require surgery, while medical management remains the choice treatment for type B dissections. Medical management consists of decreasing the blood pressure and the shearing forces of myocardial contractility in order to decrease the intimal tear and propagation of the dissection. Medical antihypertensive therapy, including beta blockers, is choice treatment.^10^

Despite progresses in diagnostic and therapeutic modalities, mortality rate is still high in aortic dissection.^8^

Abdominal Aortic Aneurysm (AAA) is the most common form of aortic aneurysms that results from degradation of medial elastin of the atheromatous aorta.^11^, ^12^

Rupture of an aortic aneurysm is life‐threatening and risk for rupture in a large aneurysm is high.^4^, ^5^ In patients with ruptured abdominal aortic aneurysm, the mortality rate is approximately 65%‐85%.^13^, ^14^ Thus, efforts have been made toward fast detection and in determining the appropriate strategy in the management of aneurysms before the rupture occurrence.

Among the various types of abdominal aortic aneurysms, isolated common iliac artery aneurysms (IAAs) are proportionately rare and the incidence is reported to range from 0.3% to 0.6%. IAAs often escape detection because of their concealed location within the pelvis and due to their leaning to be asymptomatic.^15^, ^16^

Previous studies have suggested that a cut‐off diameter above 2.4 cm could be considered the common iliac artery aneurysmal disease.^17^ The expansion rate and the rupture incidence of common IAAs are dependent on aneurysm size.^18^, ^19^ Studies recommend the asymptomatic patients with common IAAs larger than 4 cm should undergo elective repair and common IAAs larger than 5 cm should be repaired expeditiously because of their significant raised risk of rupture.^20^

The result of isolated IAAs have been reported in many investigations, but isolated common IAAs are most commonly small and the number of patients with isolated IAAs that are larger than 4 cm in diameter is generally small.^20^, ^21^

Since large isolated common IAAs are rare and there is not enough information and consensus regarding some aspects of their management, we have described a management crisis in a patient with a type B aortic dissection and a large left common iliac artery aneurysm who was at extreme high risk for complications, consequently, at the same time, we have to manage both catastrophic conditions of the aorta, making it difficult to draw a definite conclusion for this patient. Whether, initially, aortic dissection should be managed as a life threatening condition or large aortic aneurysm has been debated extensively.

According to stable hemodynamic in this patient who displayed symptomatic un ruptured AAA, we decided to delay intervention for aneurysm until aortic dissection was well managed and optimal condition was achieved. To conclude, the patient survived the risk of this double‐edge therapy.

## CONFLICT OF INTEREST

None declared.

## AUTHOR CONTRIBUTIONS

TA: analyzed data, drafted, did background research and revised the manuscript. MM: involved in patient management.

## References

[1] Shirakabe A , Hata N , Yokoyama S , et al. Diagnostic score to differentiate acute aortic dissection in the emergency room. Circ J. 2008;72:986‐990. 18503227 10.1253/circj.72.986

[2] Moon MR . Approach to the treatment of aortic dissection. Surg Clin North Am. 2009;89:869‐893. 19782842 10.1016/j.suc.2009.05.003

[3] Isselbacher EM . Epidemiology of thoracic aortic aneurysms, aortic dissection, intramular hematoma, and penetrating atherosclerotic ulcers In: Baliga RR, Nienaber CA, Isselbacher EM, Eagle KA, eds. Aortic Dissection and Related Syndromes. New York, NY: Springer Science; 2007:3‐15.

[4] Glimaker H , Holmberg L , Elvin A , et al. Natural history of patients with abdominal aortic aneurysm. Eur J Vasc Surg. 1991;5:125‐130. 2037082 10.1016/s0950-821x(05)80675-9

[5] Brown LC , Powell JT . Risk factors for aneurysm rupture in patients kept under ultrasound surveillance. UK Small Aneurysm trial participants. Ann Surg. 1999;230:289‐296; discussion 296–97. 10493476 10.1097/00000658-199909000-00002 PMC1420874

[6] Hagan PG , Nienaber CA , Isselbacher EM , et al. The international registry of acute aortic dissection (IRAD): new insights into an old disease. JAMA. 2000;283:897‐903. 10685714 10.1001/jama.283.7.897

[7] Nicholls F . Observations concerning the body of his late majesty, october 26, 1760, by Frank Nicholls, M. D. F. R. S. Physiciian to his late majesty. Philos Trans. 1761;52:265‐275.

[8] Mancini MC , Geibel J . Aortic dissection Dec 22, 2016 http://the heart.org Medscape.

[9] Criado FJ . Aortic dissection: a 250‐year perspective. Tex Heart Inst J. 2011;38(6):694‐700. 22199439 PMC3233335

[10] Tran TP , Khoynezhad A . Current management of type B aortic dissection. Vasc Health Risk Manag. 2009;5:53‐63. 19436678 PMC2672467

[11] Chaikof EL , Brewster DC , Dalman RL , et al. The care of patients with an abdominal aortic aneurysm: the Society for Vascular Surgery practice guidelines. J Vasc Surg. 2009;50:S2‐S49. 19786250 10.1016/j.jvs.2009.07.002

[12] Sakalihasan N , Limet R , Defawe OD . Abdominal aortic aneurysm. Lancet. 2005;365(9470):1577‐1589. 15866312 10.1016/S0140-6736(05)66459-8

[13] Thompson MM . Controlling the expansion of abdominal aortic aneurysms. Br J Surg. 2003;90:897‐898. 12905540 10.1002/bjs.4280

[14] Kniemeyer HW , Kessler T , Reber PU , Ris HB , Hakki H , Widmer MK . Treatment of ruptured abdominal aortic aneurysm, a permanent challenge or a waste of resources? Prediction of outcome using a multi‐organ‐dysfunction score. Eur J Vasc Endovasc Surg. 2000;19:190‐196. 10727370 10.1053/ejvs.1999.0980

[15] Lucke B , Rea MN . Studies of aneurysm. I. General statistical data on aneurysms. JAMA. 1921;77:935‐940.

[16] Lowry WF , Kraft RO . Isolated aneurysms of the iliac system. Arch Surg. 1978;113:1289‐1293. 708250 10.1001/archsurg.1978.01370230079009

[17] Armon MP , Wenham PW , Whitaker SC , Gregson RHS , Hopkinson BR . Common iliac aneurysms in patients with abdominal aortic aneurysms. Eur J Vasc Endovasc Surg. 1998;15:255‐257. 9587341 10.1016/s1078-5884(98)80186-x

[18] Desiron Q , Detry O , Sakalihasan N , Defraigne JO , Limet R . Isolated atherosclerotic aneurysms of the iliac arteries. Ann Vasc Surg. 1995;9(Suppl):S62‐S66. 8688311 10.1016/s0890-5096(06)60453-6

[19] Krupski WC , Selzman CH , Floridia R , Strecker PK , Nehler MR , Whitehill TA . Contemporary management of isolated iliac aneurysms. J Vasc Surg. 1998;28:1‐13. 9685125 10.1016/s0741-5214(98)70194-6

[20] Santilli SM , Wernsing SE , Lee ES . Expansion rates and outcomes for iliac artery aneurysms. J Vasc Surg. 2000;31:114‐121. Minneapolis, MN: Elsevier. 10642714 10.1016/s0741-5214(00)70073-5

[21] Dixon AK , Lawrence JP , Mitchell JRA . Age‐related changes in the abdominal aorta shown by computed tomography. Clin Radiol. 1984;35:33‐37. 6690178 10.1016/s0009-9260(84)80228-7

